# Prevention of breast cancer-related lymphedema by progressive resistance training – a long-term follow-up of a randomized controlled trial

**DOI:** 10.1007/s11764-026-02006-4

**Published:** 2026-04-09

**Authors:** Gunn Ammitzbøll, Marie Lillegaard, Christoffer Johansen, Charlotte Lanng, Kathrine Grell, Niels Kroman, Bo Zerahn, Ole Hyldegaard, Merete Celano Wittenkamp, Susanne Oksbjerg Dalton

**Affiliations:** 1 Survivorship Unit, Danish Cancer Institute, Strandboulevarden 49, Copenhagen, 2100 Denmark; 2 Danish Research Center for Equality in Cancer (COMPAS), Department of Clinical Oncology and Palliative Care, Zealand University Hospital, Næstved, Denmark; 3 CASTLE Late Effects Unit, Department of Oncology, Copenhagen University Hospital Rigshospitalet, Copenhagen, Denmark; 4 Department of Breast Surgery, Copenhagen University Hospital Herlev/Gentofte, Copenhagen, Denmark; 5 Statistics and Data Analysis, Danish Cancer Institute, Copenhagen, Denmark; 6 Danish Cancer Society, Copenhagen, Denmark; 7 Department of Clinical Physiology and Nuclear Medicine, Copenhagen University Hospital Herlev, Copenhagen, Denmark; 8 Section for Hyperbaric Oxygen Treatment, Department for Anaesthetics and Operations, Center for Head and Orthopaedics, Copenhagen University Hospital Rigshospitalet, Copenhagen, Denmark; 9 Department of Clinical Medicine, University of Copenhagen, Copenhagen, Denmark; 10 Department of Physiotherapy and Occupational Therapy, Copenhagen University Hospital Herlev and Gentofte, Copenhagen, Denmark; 11 Department of Sport Science and Clinical Biomechanics, University of Southern Denmark, Odense, Denmark

**Keywords:** Breast cancer–related lymphedema, Resistance training, Survivorship, Randomized controlled trial, Rehabilitation

## Abstract

**Purpose:**

The risk of breast cancer-related lymphedema (BCRL) causes much concern in survivorship, and preventive interventions are lacking. In a 3.5-year questionnaire follow-up after a randomized controlled trial, we examined prevention of BCRL by resistance training.

**Methods:**

Patients with primary unilateral breast cancer (BC) were included at surgery, randomized to usual care control (CON) or resistance training intervention (INT) commencing 2–3 weeks after surgery. The intervention comprised 20 weeks of supervised progressive resistance training followed by 30 weeks of self-administered exercise. We assessed swelling of the arm/hand, axilla or breast/chest, pain and quality of life (QOL) at baseline, 20 weeks, 1 year and 3.5 years using validated scales in mailed questionnaires.

**Results:**

Of the 158 women in the trial, 84 (53%) answered the questionnaire. At the 3.5-year follow-up, swelling was reported by 18 (62%) and 21 (39%) in the CON and INT group, respectively, yielding lower odds for the INT group (OR 0.3, 95% CI 0.09; 0.88). Effects on pain and QOL diminished over time.

**Conclusions:**

The long-term risk for self-reported BCRL was reduced for participants in early initiated resistance training compared to usual care control, for women with BC undergoing surgery, axillary lymph node dissection and radiotherapy. Limitations include a high attrition rate and studies with more complete follow-up and objective measurements are needed to confirm these results.

**Implications for cancer survivors:**

Early progressive resistance training in early physical rehabilitation after BC is safe and may be a potential long-term prevention strategy for BCRL.

**Supplementary Information:**

The online version contains supplementary material available at https://doi.org/10.1007/s11764-026-02006-4.

## Introduction

Breast cancer (BC) affects one in ten women worldwide, and with advances in screening and treatment options, survival continues to improve. In 2022, 77.279 Danish women were alive after BC, reaching a 5-year survival rate of 90.8% [1]. Therefore, the focus on improving quality of life (QOL) for survivors of BC by reducing late effects is increasingly relevant. BC related lymphedema (BCRL) causes much concern among survivors [2], being associated with pain, physical impairment, and psychological and social distress, with negative impact on QOL [3]. The risk of BCRL increases with more advanced disease and extensive treatment, including surgery with axillary lymph node dissection (ALND) and radiotherapy [4]. In addition, a few modifiable risk factors have been identified, including high body mass index (BMI) and physical inactivity [5].

The pathophysiology of BCRL is still incompletely understood, but characteristics include progressive swelling of the upper limb, shoulder or upper ipsilateral body segment, which in most cases becomes a chronic and disabling condition in need of lifelong treatment and attention [6]. Management relies on the early detection and initiation of compression combined with patient education in self-management, including risk reducing behavior, skin care and movement therapy [7].

Evidence from research in the last two decades supports the safety of resistance training both in patients at risk of BCRL and those with manifest BCRL, with positive effects on physical and psychosocial function [8, 9]. However, whether a dose response relationship exists, or whether resistance training can prevent BCRL has not been demonstrated consistently [9], and in particular long-term follow-ups of high-quality randomized studies with prevention as a primary aim are lacking [10]. We know from observational studies that the risk of BCRL is highest within the first 2 years of diagnosis, but still more than 10% of cases present after that [11, 12]. Therefore, studies with long-term follow-up on BCRL are essential for a comprehensive evaluation of the preventive effect of resistance training.

In the one-year follow-up of a randomized controlled trial (RCT) including 158 women we found no effect of resistance training on BCRL. In this study we explored the long-term effects of resistance training on BCRL, pain and QOL 3.5 years after surgery.

## Material and methods

This report describes a post-hoc study conducted as a questionnaire follow-up after the original LYCA (preventive intervention against LYmphoedema after breast CAncer) RCT.

### The LYCA – trial

The main objective in LYCA was to assess the preventive effect of an early intervention with supervised and self-administered resistance training on development of BCRL in the first year after surgery. Details about the trial, it’s results, and adherence to the intervention have been reported elsewhere [13–15]. In brief, the multicenter assessor-blinded RCT included women with primary unilateral BC and allocated participants in a 1:1 ratio stratified by BMI to an early resistance training intervention (INT) or usual care control (CON) through computer randomization. Participants were recruited and randomized on the day of surgery, baseline tested 2 weeks later, and in the following week, the INT group commenced a program of resistance training involving the major muscle groups in the upper and lower limb. The load was tailored to the individual based on the baseline strength test results and progressed gradually according to monthly testing. Supervised exercise in groups was carried out twice a week for 20 weeks in a hospital physiotherapy department, followed by self-administered exercise for 30 weeks at a self-chosen location, supported by weekly text messages. The primary outcome was BCRL measured by change in inter limb volume difference by water displacement from baseline to 1-year follow-up. Secondary outcomes were self-reported QOL and pain, assessed by validated scales at baseline, 20 weeks, and 1 year. Those who reported having BCRL at 1-year follow-up were invited to participate in an explorative study on the effect of Hyperbar oxygen therapy (HBOT). Seven and 12 participants accepted and completed treatment in the CON and INT groups, comprising 23 and 22% of the participants in the 3.5-year follow-up, respectively. Results from the study have been presented elsewhere [21], reporting no associated reduction in objectively measured BCRL at 6 months follow-up after completion of HBOT treatment.

### Long-term follow-up

From May 2019 through June 2020 follow-up questionnaires with stamped response envelopes were mailed to all participants alive, with up to two mailed and one phone call reminders to non-responders. Questionnaire items captured patient information i.e. employment status, body weight, and physical activity, the latter corresponding to national recommendations and by International Physical Activity Questionnaire (IPAQ) [16].

### Lymphedema

BCRL was assessed by self-reported presence (yes/no) of swelling of the upper quadrant (arm/hand, axilla or breast/chest), and the severity was assessed by Numeric Rating Scale (NRS) [17] with response options ranging from 0 to 10. The receipt of BCRL treatment and use of compression garments was assessed by single items. Further, BCRL-related QOL was assessed by the Lymph-ICF [18] in participants with BCRL.

### Pain

We assessed pain and its influence on daily life using a questionnaire developed for, and content validated in, a Danish nationwide breast cancer population [19]. Questions included the presence of pain in the arm/hand, axilla, or breast/chest on a weekly basis. The highest intensity of pain last week and the influence of pain on general activity, mood, work, relationships with other people, sleep, and life-enjoyment was rated from 0 to 10.

### QOL

QOL was assessed by validated global health and function scales from the European Organization for research and treatment in Cancer core module (EORTC C30) [20], and the function and symptom scales from the breast cancer specific module BR23 [21]. All scores were converted to mean scores out of 100. Further, we used the mean score for the three symptoms pain, sleeplessness and fatigue [22] from the EORTC C30 module as the symptom cluster score, and defined symptom cluster score at baseline as present if the score of each symptom was above 33. For the EORTC C30 and BR23 subscales for global health and functioning, higher score indicates a better QOL and functioning, while for symptom subscales and cluster scores a higher score indicates worse symptoms. Fatigue was assessed in further depth by the FACIT-Fatigue scale with scores ranging from 0–52, and higher score indicates less fatigue equivalent to better QOL [23].

### Sample size

The sample size was estimated according to the primary aim in the LYCA RCT. When considering > 3% increase in interlimb volume difference to be incident LE, we needed 158 participants for randomization to detect a difference of 0.43 standard deviations at 1-year follow-up with a power of 90% and statistical significance level 0.05.

### Data management

Data from returned follow-up paper questionnaires were entered into a digital database by one research assistant and validated by another, and data management was carried out by GA. A blinded statistician (KG) carried out the intention-to-treat statistical analyses. We used all data available for each measurement timepoint in mixed effect models.

### Statistical analyses

Categorical variables measured at one time point were compared between groups with Fisher’s exact test. We assessed the effect of the intervention on BCRL and any pain last week as well as the intensity of both BCRL, pain, and pain influence using generalized mixed-effects models with possible correlation between measures from the same person included using a compound symmetry or unstructured covariance matrix for the binary and continuous outcomes, respectively. We assumed the two groups were similar at baseline due to the randomization; hence, we fitted models with a common intercept. Moreover, we included an interaction between the visit and allocated group at each time point allowing for a different intervention effect at the follow-up assessments. The overall effect of allocated group was tested with a chi-square test comparing the model with and without the group variable.

The effect of intervention on the QOL scales were assessed using a tobit mixed model assuming no difference between groups and an interaction between group and visit at the three follow-up assessments. Possible correlation was accounted for using an unstructured covariance matrix. The pre-defined subgroup analyses examining the effect in those with/without the symptom cluster present at baseline were performed by adding an interaction term between allocation group and presence of symptom cluster to the model. All analyses were carried out using R version 4.2.0, and p-values < 0.05 were considered statistically significant.

## Results

**Fig. 1:**
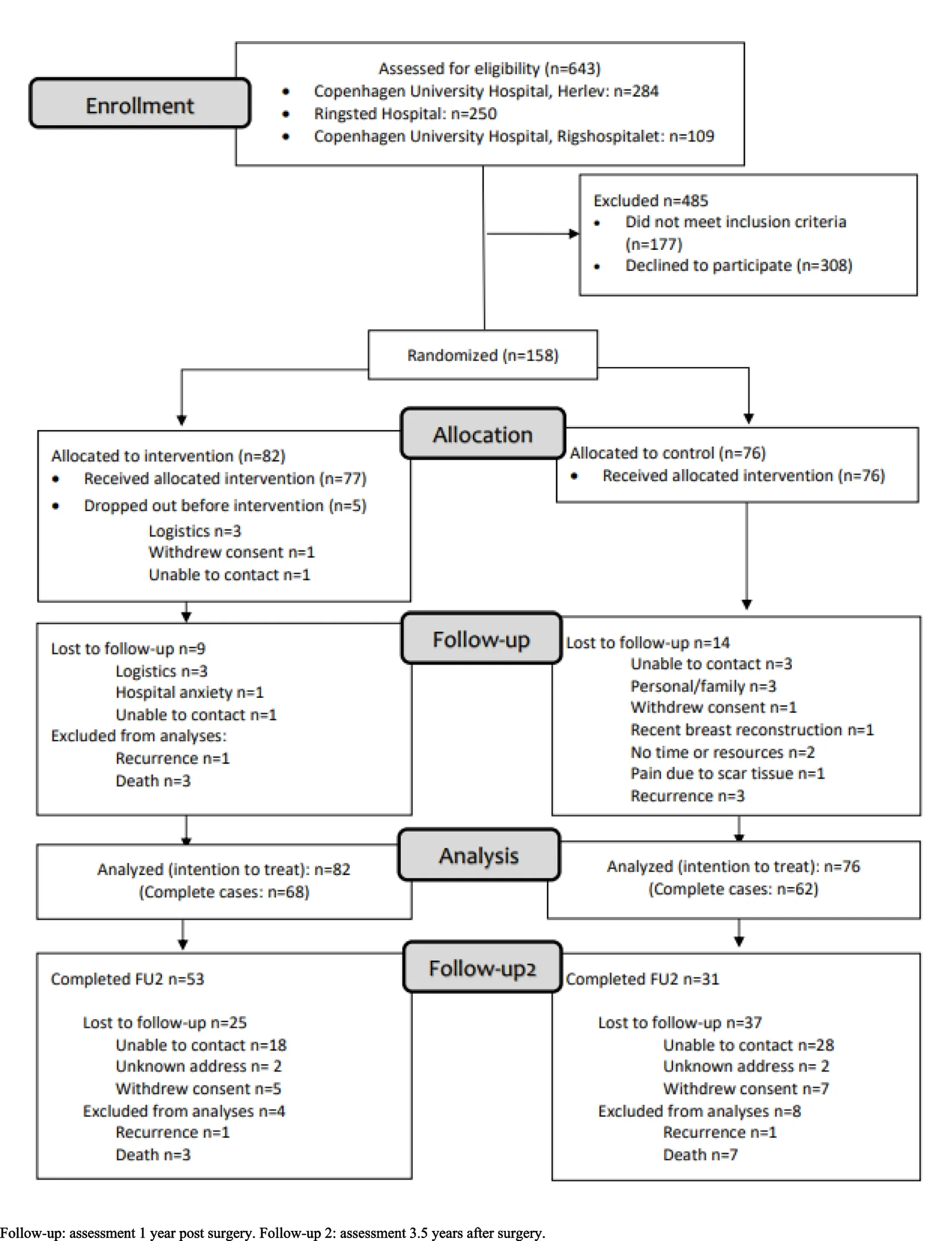
Recruitment and participation at 1, and 3.5-year follow-up of 158 women treated for primary breast cancer with axillary lymph node dissection, LYCA study, 2015–2022, East Denmark

Out of the 158 women who entered the RCT, 84 (53%) participated in the 3.5-year follow-up (Fig. 1).

The main reason for not participating in the 3.5-year follow-up was non-response to contact attempts. Table 1 shows participants’ baseline characteristics for all participants and stratified for participation in the 3.5-year follow-up. Mean time from surgery to final questionnaire was 3.5 years (standard deviation [SD] 0.36). At 3.5-years follow-up, 3 (10%) and 24 (44%) of participants in the CON and INT groups, respectively, were part-time employed with wage subsidies, while none were full-time employed. The mean BMI increased for both groups from baseline to 3.5-years follow-up, with 1.1 kg (SD 5.1) for the CON group and 2.6 kg (SD 5.6) for the INT group, and with the increase taking place mainly after the first year (data not shown). At 3.5-years follow-up, there was no statistically significant difference between physical activity in the two groups, with light to moderate physical activity above 30 min/day reported by 22 (71%) and 40 (74%) in the CON and INT groups, respectively (data not shown).

**Table 1:** Baseline clinical, demographic and lifestyle characteristics for all participants, stratified by participation and non-participation in 3.5-year follow-up in the LYCA study, 2015–2022, East Denmark

Group allocation		All participants		Participants in 3.5-year FU		Participants lost to 3.5-year FU	
		C (n = 76)	I (n = 82)	C (n = 31)	I (n = 53)	C (n = 45)	I (n = 29)
Age in years, mean (SD)		52 (10)	53 (10)	52 (9)	55 (10)	53 (11)	50 (10)
Living arrangement, n (%)	Cohabiting	48 (63)	65 (79)	25 (81)	41 (77)	23 (51)	24 (86)
	Living alone	20 (26)	15 (18)	6 (19)	12 (23)	14 (31)	3 (11)
	Missing	8 (11)	2 (2)	0	1	8 (18)	1 (4)
Education, n (%)	Mandatory school	3 (4)	6 (7)	1 (3)	5 (9)	2 (44)	1 (4)
	Secondary high school	7 (9)	7 (9)	2 (7)	6 (11)	5 (11)	1 (4)
	Higher education, Short	21 (28)	12 (15)	9 (29)	8 (15)	12 (27)	4 (14)
	Higher education, Medium	26 (34)	37 (45)	12 (39)	24 (44)	14 (31)	13 (46)
	Higher education, Long	11 (15)	17 (21)	7(23)	10 (19)	4 (9)	7 (25)
	Missing	8	3	0	1	8 (18)	2 (7)
Employment at diagnosis, n (%)	Full- or part-time	53 (70)	63 (77)	26 (84)	41 (76)	27 (69)	22 (79)
	Other	15 (20)	17 (21)	5 (16)	12 (22)	10 (22)	5 (18)
	Missing	8 (11)	2 (2)	0	1 (2)	8 (18)	1 (4)
Dominant limb affected, n (%)		40 (53)	38 (46)	16 (52)	23 (43)	24 (53)	15 (54)
Surgery n (%)	Lumpectomy	41 (54)	43 (52)	20 (65)	31 (57)	21 (47)	12 (43)
	Mastectomy	35 (46)	39 (52)	11 (36)	23 (43)	24 (53)	16 (57)
Tumor diameter in mm, mean (SD)		23 (12)	22 (12)	23 (11)	20 (11)	23 (14)	25 (13)
Lymph nodes extracted, mean (SD)		19 (8)	19 (9)	20 (7)	19 (10)	18 (9)	20 (6)
Positive lymph nodes, mean (SD)		3 (3)	3 (4)	3 (4)	2 (3)	3 (3)	4 (5)
Hist. stage of malignancy n (%)	1	16 (21)	9 (11)	6 (19)	8 (15)	10 (22)	1 (4)
	2	31 (41)	46 (56)	15 (48)	30 (56)	16 (36)	16 (57)
	3	16 (21)	15 (18)	6 (19)	9 (17)	10 (22)	6 (21)
	Missing	13 (17)	12 (15)	4 (13)	7 (13)	9 (20)	5 (18)
ER positive, n (%)		54 (71)	69 (84)	23 (74)	47 (87)	31 (69)	22 (79)
Hormone treatment, n (%)		51 (67)	64 (78)	23 (74)	46 (85)	28 (62)	18 (64)
	Missing	2 (3)	0	0	0	2 (4)	0
Chemotherapy n (%)	Adjuvant	45 (60)	48 (59)	19 (61)	26 (48)	26 (58)	22 (79)
	Neoadjuvant	20 (27)	25 (30)	9 (29)	20 (37)	12 (27)	5 (18)
	No chemotherapy	10 (13)	9 (11)	3 (10)	8 (15)	7 (16)	1 (4)
BMI mean (SD), kg/m^2^		27 (4,5)	26 (5,6)	27 (4,1)	26 (5,2)	26 (4,9)	25 (6,4)
BMI, n (%)	≤ 25 kg/m^2^	29 (38)	42(51)	12 (39)	26 (48)	20 (44)	16 (57)
	> 25 to ≤ 30 kg/m^2^	24 (31)	18 (22)	12 (39)	12 (22)	12 (27)	6 (21)
	> 30 to ≤ 35 kg/m^2^	8 (11)	15 (18)	5 (16)	11 (20)	3 (7)	4 (14)
	> 35 kg/m^2^	5 (7)	3 (4)	2 (7)	2 (4)	3 (7)	1 (4)
	Missing	10 (13)	4 (5)	0	3 (6)	10 (22)	1 (4)
Physical activity, n (%)	Inactive	4 (6)	0	2 (7)	0	2 (4)	0
	< 30 min./day	22 (32)	30 (38)	13 (42)	19 (35)	9 (20)	11 (39)
	≥ 30 min./day	22 (32)	32 (40)	7 (23)	21 (39)	15 (33)	11 (39)
	≥ 30 min/day + HIT	20 (29)	18 (22,5)	9 (29)	13 (24)	11 (24)	5 (18)
	Missing	8 (11)	2 (2)	0	1 (2)	8 (18)	1 (4)
Smoking, n (%)	Current smoker	5(7)	4 (5)	2 (7)	28 (52)	3 (7)	2 (7)
	Former smoker	34 (45)	34 (42)	17 (55)	23 (43)	17 (38)	11 (39)
	Never smoker	26 (34)	43 (52)	11 (36)	28 (52)	15 (33)	15 (54)
	Missing	11 (15)	1 (1)	1 (3)	1 (2)	10 (22)	0
Alcohol units/week	Mean (SD)	4 (4)	5 (6)	5 (5)	5 (5)	3 (4)	5 (6)
	No intake, n (%)	22(29)	22 (27)	10 (32)	15 (28)	12 (27)	7 (25)

C control group, I intervention group, n number, FU follow-up, Education: Primary, primary school only, General/technical, higher general or technical exam; Higher-Short short-cycle higher education, Higher-Medium mediumcycle higher education, Higher-Long, long-cycle higher education. Employment: Unemployed/other, unemployed/pensioned/sick leave/other. SD standard deviation. Physical activity (self-reported): HIT, high Iintensity training at recommended levels, corresponding to ≥ 30 min twice/week. ER estrogen receptor, HER2 human epidermal growth factor receptor 2, Hist histological

### Lymphedema

At 3.5-year follow-up 4 (13%) in the CON group and 4 (7%) in the INT group reported currently receiving BCRL treatment, and 14 (45%) in the CON group and 19 (35%) in the INT group currently used compression ≥ 1/week. Swelling at 3.5-year follow-up was reported by 18 (62%) in the CON group, and 21 (39%) in the INT group yielding a lower OR for reporting swelling for INT versus CON (OR 0.27, 95% confidence interval [CI] 0.09; 0.88) (Table 2). In those who reported having swelling, we found no overall difference in the mean intensity score across all time points between groups (p = 0.07) (data not shown).

**Table 2:** Effect of the intervention on the presence of self-reported swelling in women with primary breast cancer treated surgically with axillary lymph node dissection and radiotherapy the LYCA study, 2015–2022, East Denmark

**Assessment time**				**OR for self-reported swelling for the INT compared with CON group**		
		Control	Intervention	OR	95% CI	p-value
Baseline	Yes, n	54	57			
	No, n	13	23			
	% yes	81%	71%			
20-week FU	Yes, n	37	34	1.10	0.42; 2.88	0.841
	No, n	21	19			
	% yes	63%	29%			
12-month FU	Yes, n	41	38	0.56	0.22; 1.43	0.224
	No, n	17	25			
	% yes	71%	60%			
3.5-year FU	Yes, n	18	21	0.27	0.09; 0.88	**0.029**
	No, n	11	33			
	% yes	62%	39%			

FU: Follow-up. n: number OR: odds ratio. CON: control group. INT: intervention group. Bold numbers indicate statistical significance (p<0,05). 95% CI: 95% confidence interval

**Fig. 2:**
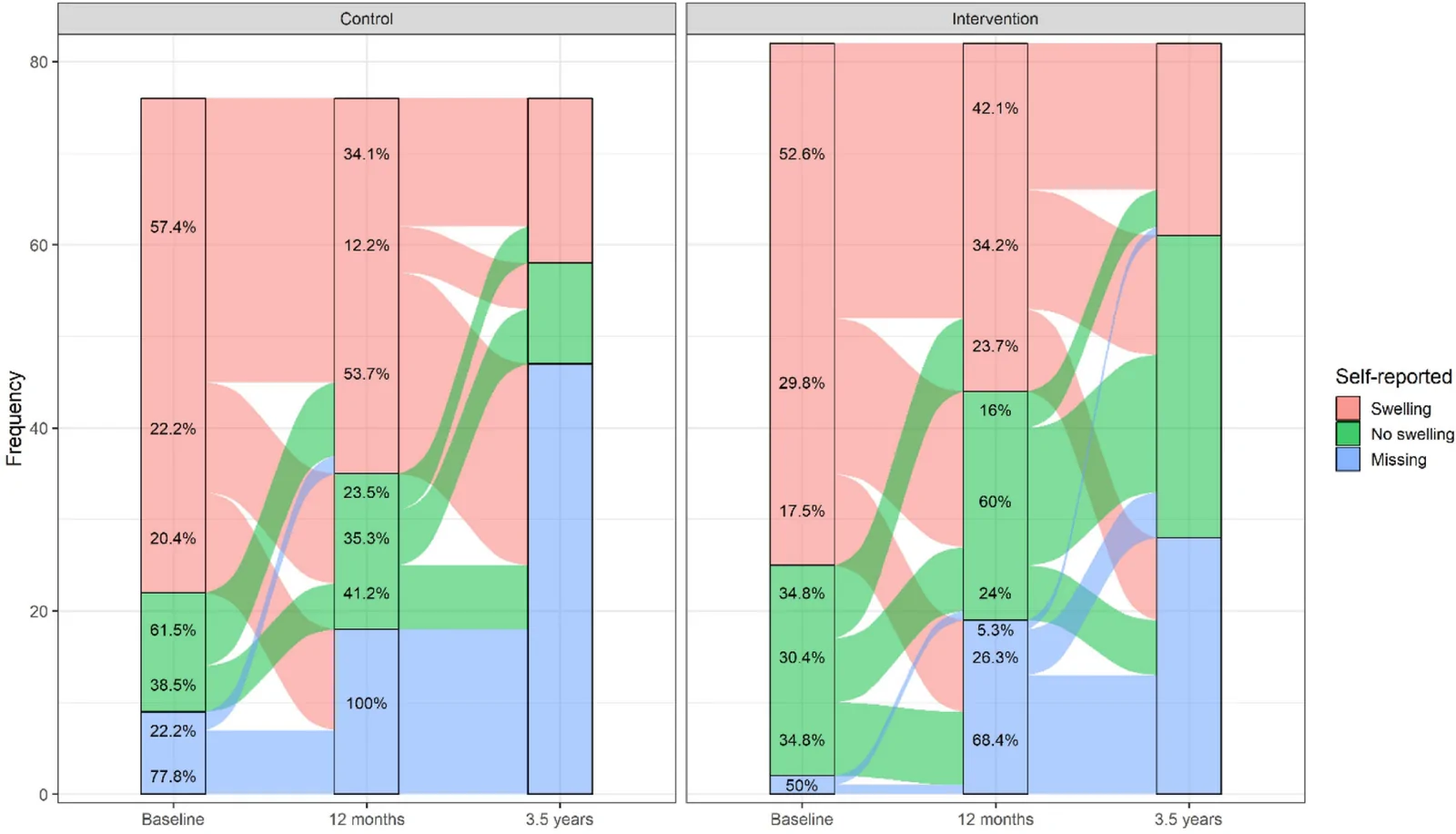
Self-reported swelling status change from baseline to 1-year and 3.5 -year follow-up for participants in the LYCA study, 2015–2022, East Denmark

The change of swelling status in the study period (yes/no/missing) is shown in Fig. 2. In the CON group, 24% developed swelling from 12-month follow-up to 3.5-year follow-up and 12% experienced a reverse of the swelling compared to 16% and 34%, respectively, in the INT group. However, at 3.5-year follow-up, self-reported swelling was missing for 54% and 24% for the CON and INT groups, respectively (Fig. 2).

Y-axis: frequency of self-reported swelling yes/no/missing. X-axis: time of measurement baseline, 12 months post surgery and 3,5 years post surgery. Percentages in the columns indicate the percentage of participants in each category which transitions to either swelling, no swelling or missing for the next measurement time point, according to the adjacent color and flow to the right of the column. Control: usual care control group. Intervention: progressive resistance training intervention group.

### Pain 

The OR for reporting pain within the last week was similar between groups through the entire intervention (p = 0.85; Supplementary Table 1), and the mean pain intensity was not different between the groups at 3.5-year follow-up (p = 0.55; Supplementary Fig. 1).

We found a small but significant effect on the influence of pain on the relationship with other people with 0.5 (95% CI: 0.1; 1.0) and 0.7 (95% CI: 0.2; 1.2) points lower mean score for the INT group at 20-week and 12-month follow-up, (p = 0.02 and p = 0.004, respectively) but not at 3.5 years (p = 0.20). We found no effect of the intervention on the other aspects of daily life (influence of pain on general physical activity, mood, work, sleep and enjoyment of life) (Supplementary Fig. 2).

### Quality of life

QOL measured with the EORTC C30 function scales and BR23 function and symptom scales showed no consistent findings of a long-term effect of the intervention (Supplementary Table 2). We did find small overall effects on emotional, social and sexual functioning across all time points, but the effects diminished over time.

For fatigue, we found no effect of the intervention at 3.5-year follow-up, nor was there an effect on the symptom cluster score (pain-sleeplessness-fatigue) (Supplementary Table 2). In subgroup analyses, we found an effect for global health, emotional, cognitive, and social functioning in those with a symptom cluster present at baseline, whereas for the group with no symptom cluster, we only found an effect on emotional functioning (Supplementary Table 3). When testing for an interaction between presence of symptom cluster at baseline and EORTC function scales at all time points, we found a significant effect for global health and social functioning (Supplementary Table 3).

## Discussion

For the first time, in a long-term follow-up of a randomized controlled trial examining the effect of early initiated resistance training after breast cancer surgery, our results suggest that resistance training causes fewer to experience swelling in the long term compared to usual care. We did not see this effect at earlier follow-ups. We found that the modest tendencies of effect on pain and QOL at 12-month follow-up had diminished at 3.5-year follow-up, except for better overall social and emotional functioning, which persisted across all time points. This was especially apparent for participants who presented with the symptom cluster pain-sleeplessness-fatigue at baseline. As attrition was high, especially in the CON group, we emphasize that all results should be interpreted with caution.

### Lymphedema

Our finding suggesting a long-term effect on self-reported swelling among those who participated in progressive resistance training stands in contrast to our finding at 12-month follow-up, where there was no effect of the intervention on inter limb volume difference or self-reported symptoms [14]. Self-reported swelling is considered a more sensitive measure of BCRL, returning higher prevalences than water displacement [24], and it covers the important personal experience of the disease. However, in recent research, the triangulation of measurement methods has been advocated, as there is no consensus to one single method being superior to the others, and rather, they capture different properties of the disease. We asked participants to report swelling in the whole upper quadrant (arm/hand, axilla, breast and thorax), which, while including more of the common patient-experienced features of BCRL, contributes to inflated estimates compared to i.e. arm volume measurements alone [25]. Previous studies have also indicated long-term effects of resistance training on BCRL in subgroups. One of the first high-quality studies to report this was the study by Schmitz et al. [26], who found that those with more than five lymph nodes removed, less frequently had BCRL at 1-year follow-up when participating in resistance training. However, this population had a mean time since surgery of 39 months and had not experienced BCRL at study entry, which indicates they may have a low-risk profile. More recently, systematic literature reviews and meta-analyses have shown that resistance training may have a role in prevention of BCRL [27, 28]. Suggested mechanisms include increased pumping of skeletal muscles enhancing the return of lymph into collective ducts and the blood flow. Further, the effect of resistance training on low grade systemic inflammation may be included in the chain of effects [29, 30]. Physical inactivity and high BMI are well-documented risk factors for BCRL [4, 5] and considering the link between systematic and maintained exercise habits and BMI, these may also be explanatory factors. In this study there was no difference between groups in level of physical activity, but more detailed and objective information about type of exercise and body composition could have informed findings and provided hypotheses about mechanisms involved.

### Pain

We did not find an effect of the intervention on the long-term experience of pain, despite a tendency of a favorable effect on pain outcomes within the first year after surgery, with the largest difference between groups in the short term (20 weeks post-surgery) and diminishing towards the 1-year follow-up. Similar results were found in a small study on the analgesic effect of resistance training in patients with persistent pain after BC, where the effect had diminished 3 months after the intervention [31].

These findings agree with a recent systematic review and network meta-analysis including 3003 patients with breast cancer reporting on seven exercises modes. The authors found no statistically significant effect on pain, although the results tended to favor participants in combined aerobic and resistance exercise interventions [32]. Other recent systematic reviews have found small effects on pain in studies of exercise in patients across cancer types [33–35] and in studies of exercise for the prevention and treatment of BCRL [9]. Taken together, the results are not sufficient to provide precise exercise prescriptions in terms of frequency, intensity, time and type required, as was also the conclusion by an international multidisciplinary roundtable on exercise guidelines for cancer survivors in 2019 [36]. Notably, the available knowledge largely relies on studies with follow-up until 1–2 years, although pain for women with or at risk of BCRL has been documented to persist up to 10 years post-diagnosis [37], indicating an evidence gap.

### QOL and fatigue

Our findings suggest no consistent effect of the intervention on QOL and fatigue outcomes, except for a tendency of better overall social, emotional and sexual functioning in the long-term. In general, participants in both groups reported high levels of functioning and low levels of symptoms during the study period, leaving little potential for improvement or prevention. In the subgroup analysis examining the effect for those with the cluster pain-sleeplessness-fatigue at baseline, we did see a favorable effect of the intervention on several QOL domains. Our results are partly in line with recent systematic reviews in BC. In a network meta-analysis Dong et al. [32] found an effect of combined resistance and aerobic training on QOL but not fatigue, which was confirmed by Zhang et al. [38] in a systematic review including 21 randomized controlled trials. In studies specifically examining the preventive effect of exercise on LE, Hayes and colleagues [9] found statistically significant improvements both for QOL and fatigue. Although effects are consistently documented, the studies vary in terms of domains examined, intervention dose and delivery, as well as survivorship phase. Further, most studies are limited to 1–2 years of follow-up after diagnosis. The OptiTrain study (n = 240) is one of the few studies reporting on follow-up beyond 2 years [39] in patients with breast cancer who participated in either resistance- or aerobic training compared with usual care. The results of the 5-year follow-up stand in contrast to the literature reviews, finding no effect on QOL outcomes or on fatigue. The long-term effect of exercise on QOL evidently needs further attention to provide precise targeted exercise prescriptions.

### Strengths and limitations

This study has a substantial loss to follow-up. Explanations may include that, increasing with time, survivors may wish to leave disease and treatment behind and not be reminded of that period in their lives. Additionally, more than 2 years with no contact from the project group may reduce the feeling of obligation to respond to contact attempts. Moreover, patients with poorer outcomes may well be more reluctant to provide feedback. We provide a thorough overview of baseline characteristics between participants and non-participants in the 3.5-year follow-up for transparency regarding the loss to follow-up and to provide perspectives for interpretation of the results. We chose not to conduct any sensitivity analyses using multiple imputations, as this did not considerably change the results in our previous paper [13].

We used self-report measurement of BCRL as a low budget method, convenient for participants in hope of a higher response rate. However, self-report may also have its relevance as an overarching patient-centered evaluation, as research in BCRL measurement is still advancing and no objective physical method completely covers all aspects of the disease, although we acknowledge that the nature of the self-report data also do not necessarily reflect the women’s full experience with BCRL. Importantly, studies have found self-report to overestimate prevalence [40] but be useful in prediction of BCRL, being closely associated with objective measures of its’ physical features [41]. Self-report may, however, have introduced bias with higher self-reported symptoms in the control group in a subconscious response to not receiving the preventative intervention, which may have been further exaggerated by higher drop-out rate in the control group. Finally, we did not specifically measure adherence to resistance training in the follow-up beyond one year, which could have added substance to our discussion of the findings.

In this post-hoc study, the sample size was not powered to detect a difference in this long-term follow-up. Moreover, the risk of chance findings must be considered, as we have performed a high number of statistical tests; hence, we emphasize that all results should be interpreted with caution.

## Conclusions

In this long-term follow-up of the LYCA study, we found indications that women with BC at high risk for LE participating in resistance training experienced less swelling in the long term, although this benefit was not apparent within the first post-operative year. Additionally, the initial favorable tendencies in pain, QOL and fatigue appeared to diminish over time. Notably, we found that women presenting with a pain-sleeplessness-fatigue cluster at baseline, may derive particular benefit from participation in a structured resistance exercise program. Given the study’s limitations, these findings should be interpreted with caution, and they do not, on their own, justify changes to clinical practice recommendations. Importantly, however, our findings support the evidence underpinning the safety of participating in resistance training after breast cancer treatment, even for those at elevated risk of BCRL.

Future research should prioritize more complete long-term follow-up, objective measurements of BCRL, and detailed evaluation of adherence to exercise interventions to elucidate the preventive potential of resistance exercise. Moreover, to ensure that patients with cancer can benefit from exercise throughout the cancer continuum, investment in implementation is essential to facilitate the integration of structured exercise, including resistance training, into routine cancer care.

## Supplementary Information

Below is the link to the electronic supplementary material.

ESM 1 (DOCX 64.7 KB)

ESM 2 (DOCX 139 KB)

ESM 3 (DOCX 17.6 KB)

ESM 4 (DOCX 30.2 KB)

ESM5 (DOCX 21.1 KB)

## Data Availability

From authors upon reasonable request.
