# Supplementary material for: Prevention of breast cancer-related lymphedema by progressive resistance training – a long-term follow-up of a randomized controlled trial
Source: J Cancer Surviv. 2026 Apr 9;20(Suppl 2):1834–44. doi: 10.1007/s11764-026-02006-4 (PMC13744473; doi:10.1007/s11764-026-02006-4)
Supplement: Supplementary file 1 [file 11764_2026_2006_MOESM1_ESM.docx]

**Prevention of breast cancer-related lymphedema by progressive resistance training – a long-term follow-up of the LYCA Randomized Controlled Trial.**

**Journal of Cancer Survivorship**

Gunn Ammitzbøll, Marie Lillegaard, Christoffer Johansen, Charlotte Lanng, Kathrine Grell, Niels Kroman, Bo Zerahn, Ole Hyldegaard, Merete Celano Wittenkamp, Susanne Oksbjerg Dalton.

**Corresponding author**:

Gunn Ammitzbøll

Danish Cancer Institute, Strandboulevarden 49, 2100 Copenhagen, Denmark. Email: gunnam@cancer.dk.

The vertical lines show 95% confidence intervals.

Supplementary Figure 1. Estimated mean level for worst pain intensity experienced in the last week (0–10) for participants in the LYCA study, 2015–2022, East Denmark.
