# Supplementary material for: Prevention of breast cancer-related lymphedema by progressive resistance training – a long-term follow-up of a randomized controlled trial
Source: J Cancer Surviv. 2026 Apr 9;20(Suppl 2):1834–44. doi: 10.1007/s11764-026-02006-4 (PMC13744473; doi:10.1007/s11764-026-02006-4)
Supplement: Supplementary file 3 [file 11764_2026_2006_MOESM3_ESM.docx]

**Prevention of breast cancer-related lymphedema by progressive resistance training – a long-term follow-up of the LYCA Randomized Controlled Trial.**

**Journal of Cancer Survivorship**

Gunn Ammitzbøll, Marie Lillegaard, Christoffer Johansen, Charlotte Lanng, Kathrine Grell, Niels Kroman, Bo Zerahn, Ole Hyldegaard, Merete Celano Wittenkamp, Susanne Oksbjerg Dalton.

**Corresponding author**:

Gunn Ammitzbøll

Danish Cancer Institute, Strandboulevarden 49, 2100 Copenhagen, Denmark. Email: gunnam@cancer.dk.

**Supplemetary Table 1:** Effect of the intervention on the presence of pain last week in women with primary breast cancer treated surgically with axillary lymph node dissection and radiotherapy the LYCA study, East Denmark.

| Assessment | |  | | OR for INT compared to the CON group | | |  |
| --- | --- | --- | --- | --- | --- | --- | --- |
|  |  | **CON** | **INT** | **OR** | **95% CI** | **p-value** | **Overall p-value** |
| Baseline | Yes, n | 58 | 66 |  | | | 0.854**^a^** |
|  | No, n | 10 | 14 |  |  |  |  |
|  | % yes | 85% | 83% |  |  |  |  |
| 20-week FU | Yes, n | 35 | 30 | 0.78 | 0.25; 2.47 | 0.676 |  |
|  | No, n | 23 | 24 |  |  |  |  |
|  | % yes | 60% | 56% |  |  |  |  |
| 12-month FU | Yes, n | 36 | 42 | 1.12 | 0.37; 3.43 | 0.838 |  |
|  | No, n | 22 | 24 |  |  |  |  |
|  | % yes | 62% | 64% |  |  |  |  |
| 3.5-year FU | Yes, n | 15 | 27 | 0.61 | 0.15; 2.42 | 0.479 |  |
|  | No, n | 15 | 27 |  |  |  |  |
|  | % yes | 50% | 50% |  |  |  |  |

FU: Follow-up. n: number. BCRLE: breast cancer related lymphedema. OR: odds ratio. CON: control group. INT: intervention group. p: p-value. 95% CI: 95% confidence interval. **^a^** Test of between group differences across all assessment times.
