# Supplementary material for: Prevention of breast cancer-related lymphedema by progressive resistance training – a long-term follow-up of a randomized controlled trial
Source: J Cancer Surviv. 2026 Apr 9;20(Suppl 2):1834–44. doi: 10.1007/s11764-026-02006-4 (PMC13744473; doi:10.1007/s11764-026-02006-4)
Supplement: Supplementary file 4 [file 11764_2026_2006_MOESM4_ESM.docx]

**Prevention of breast cancer-related lymphedema by progressive resistance training – a long-term follow-up of the LYCA Randomized Controlled Trial.**

**Journal of Cancer Survivorship**

Gunn Ammitzbøll, Marie Lillegaard, Christoffer Johansen, Charlotte Lanng, Kathrine Grell, Niels Kroman, Bo Zerahn, Ole Hyldegaard, Merete Celano Wittenkamp, Susanne Oksbjerg Dalton.

**Corresponding author**:

Gunn Ammitzbøll

Danish Cancer Institute, Strandboulevarden 49, 2100 Copenhagen, Denmark. Email: gunnam@cancer.dk.

**Supplementary Table 2.** Effect of progressive resistance training on quality of life and fatigue in women with primary breast cancer treated with axillary lymph node dissection and radiotherapy the LYCA study, 2015–2022, East Denmark.

|  | Assessment time | Control Intervention | | | | Difference between INT and CON | 95% CI | p-value | Overall p-value^a^ |
| --- | --- | --- | --- | --- | --- | --- | --- | --- | --- |
|  |  | n | Mean score (sd) | n | Mean score (sd) |  |  |  |  |
| EORTC Quality of life C30 module, function scales (higher score is better) | | | | | | | | | |
| Global Health | 20 w | 59 | 69 (21) | 54 | 72 (18) | 1.9 | -5.5; 9.2 | 0.60 | 0.19 |
|  | 12 m | 58 | 73 (21) | 67 | 78 (18) | 6.0 | -1.2; 13.1 | 0.09 |  |
|  | 3.5 y | 31 | 75 (21) | 53 | 72 (20) | -4.6 | -13.4; 4.1 | 0.30 |  |
| Physical Function | 20 w | 59 | 89 (12) | 55 | 90 (11) | 3.9 | -2.6; 10.4 | 0.23 | 0.46 |
|  | 12 m | 58 | 87 (16) | 67 | 90 (16) | 3.1 | -3.3; 9.3 | 0.34 |  |
|  | 3.5 y | 30 | 86 (16) | 54 | 85 (17) | -0.6 | -8.3; 6.7 | 0.87 |  |
| Role Function | 20 w | 58 | 78 (28) | 54 | 81 (24) | 1.9 | -12.8; 16.3 | 0.80 | 0.93 |
|  | 12 m | 58 | 81 (24) | 67 | 86 (23) | 10.3 | -4.1; 24.3 | 0.15 |  |
|  | 3.5 y | 30 | 78 (31) | 54 | 83 (25) | 4.3 | -13.4; 21.6 | 0.63 |  |
| Emotional Function | 20 w | 59 | 78 (22) | 55 | 89 (13) | 9.1 | 1.1; 17.0 | **0.02** | **<0.001** |
|  | 12 m | 58 | 80 (22) | 67 | 88 (15) | 8.8 | 1.0; 16.5 | **0.02** |  |
|  | 3.5 y | 31 | 91 (12) | 53 | 85 (18) | -10.1 | -19.9; -0.5 | **0.04** |  |
| Cognitive Function | 20 w | 59 | 71 (29) | 55 | 80 (21) | 5.7 | -5.7; 16.9 | 0.32 | 0.11 |
|  | 12 m | 58 | 75 (25) | 67 | 85 (21) | 12.4 | 1.2; 23.4 | 0.03 |  |
|  | 3.5 y | 31 | 82 (22) | 53 | 82 (25) | 4.3 | -9.3; 17.7 | 0.53 |  |
| Social Function | 20 w | 59 | 75 (29) | 55 | 89 (17) | 16.5 | 1.7; 30.9 | **0.03** | **0.02** |
|  | 12 m | 58 | 82 (26) | 67 | 88 (20) | 11.8 | -2.7; 25.9 | 0.10 |  |
|  | 3.5 y | 31 | 87 (26) | 53 | 86 (23) | -2.2 | -20.4; 15.6 | 0.81 |  |
| Breast Cancer specific module BR23, function Scales (higher score is better) | | | | | | | | | |
| Body Image | 20 w | 59 | 75 (27) | 55 | 79 (20) | 2.6 | -6.8; 11.9 | 0.58 | 0.41 |
|  | 12 m | 58 | 82 (21) | 67 | 84 (20) | 3.7 | -5.6; 12.7 | 0.43 |  |
|  | 3.5 y | 30 | 80 (23) | 54 | 81 (21) | 8.1 | -3.0; 19.0 | 0.15 |  |
| Sexual Function | 20 w | 57 | 17 (20) | 55 | 25 (22) | 8.2 | 1.4; 14.8 | **0.02** | **<0.001** |
|  | 12 m | 55 | 19 (20) | 66 | 27 (24) | 10.1 | 3.5; 16.5 | **<0.001** |  |
|  | 3.5 y | 29 | 27 (23) | 53 | 25 (22) | -0.1 | -8.1; 7.8 | 0.99 |  |
| Sexual enjoyment | 20 w | 22 | 53 (20) | 31 | 54 (25) | 4.6 | -11.8; 20.8 | 0.57 | 0.37 |
|  | 12 m | 24 | 57 (39) | 38 | 64 (24) | 9.1 | -6.7; 24.6 | 0.25 |  |
|  | 3.5 y | 17 | 65 (28) | 30 | 58 (23) | -6.8 | -24.9; 11.0 | 0.45 |  |
| Future perspective | 20 w | 59 | 53 (30) | 55 | 59 (28) | 3.8 | -6.9; 14.2 | 0.48 | 0.06 |
|  | 12 m | 58 | 53 (28) | 67 | 63 (25) | 8.6 | -1.8; 18.6 | 0.10 |  |
|  | 3.5 y | 30 | 64 (29) | 54 | 58 (32) | -9.3 | -22.2; 3.2 | 0.15 |  |
| Breast Cancer specific module, symptom scales (higher score is worse) | | | | | | | | | |
| Systemic therapy side effects | 20 w | 59 | 25 (22) | 55 | 22 (16) | 2.3 | -3.2; 8.2 | 0.38 | 0.83 |
|  | 12 m | 58 | 17 (14) | 67 | 17 (12) | -0.4 | -9.0; 5.1 | 0.89 |  |
|  | 3.5 y | 30 | 17 (12) | 54 | 18 (13) | -0.9 | -7.9; 5.9 | 0.80 |  |
| Breast symptoms | 20 w | 59 | 14 (14) | 55 | 17 (15) | -3.6 | -10.8; 3.5 | 0.32 | 0.16 |
|  | 12 m | 58 | 20 (19) | 66 | 15 (12) | 4.5 | -2.4; 11.3 | 0.19 |  |
|  | 3.5 y | 30 | 12 (16) | 54 | 12 (11) | -0.7 | -9.6; 8.1 | 0.88 |  |
| Arm Symptoms | 20 w | 59 | 20 (18) | 55 | 18 (16) | -0.9 | -9.0; 7.1 | 0.83 | 0.26 |
|  | 12 m | 58 | 24 (22) | 66 | 21 (21) | 1.1 | -6.8; 8.7 | 0.79 |  |
|  | 3.5 y | 30 | 25 (22) | 54 | 18 (21) | 9.4 | -0.4; 19.0 | 0.06 |  |
| Upset by Hair Loss | 20 w | 19 | 60 (44) | 17 | 63 (46) | 5.7 | -22.7; 33.6 | 0.69 | 0.95 |
|  | 12 m | 4 | 67 (33) | 7 | 52 (33) | 11.1 | -34.3; 55.6 | 0.63 |  |
|  | 3.5 y | 9 | 56 (33) | 18 | 50 (33) | 4.1 | -27.7; 35.4 | 0.80 |  |
| FACIT-fatigue scale (higher score is better) | | | | | | | | | |
| Fatigue | 20 w | 59 | 38 (10) | 55 | 41 (8) | 2.78 | -0.04; 5.55 | **0.05** | 0.12 |
|  | 12 m | 57 | 37 (11) | 66 | 43 (7) | 2.33 | -0,42;5.03 | 0.09 |  |
|  | 3.5 y | 30 | 41 (10) | 53 | 42 (10) | 0.44 | -2.92; 3.73 | 0.79 |  |
| Symptom Cluster score (pain – sleeplessness – fatigue) (higher score is worse) | | | | | | | | | |
| Symptom cluster score | 20 w | 59 | 31 (22) | 55 | 23 (17) | -4.55 | -10.9; 1.71 | 0.15 | 0.52 |
|  | 12 m | 58 | 30 (26) | 67 | 25 (20) | -2.37 | -8.53; 3.68 | 0.44 |  |
|  | 3.5 y | 30 | 25 (24) | 54 | 25 (23) | 0.02 | -7.53; 7.41 | >0.99 |  |

CON: control group. INT: intervention group. CI: confidence interval. Sd: standard deviation. EORTC: European Organization for Research and Treatment of Cancer. C30: core 30-item scale. BR23: Breast cancer specific module of the EORTC. FACIT: Functional Assessment of Chronic Illness Therapy. Bold numbers indicate p-value < 5%. **^a^** Test of between group differences across all assessment times.
