# Supplementary material for: Prevention of breast cancer-related lymphedema by progressive resistance training – a long-term follow-up of a randomized controlled trial
Source: J Cancer Surviv. 2026 Apr 9;20(Suppl 2):1834–44. doi: 10.1007/s11764-026-02006-4 (PMC13744473; doi:10.1007/s11764-026-02006-4)
Supplement: Supplementary file 5 [file 11764_2026_2006_MOESM5_ESM.docx]

**Prevention of breast cancer-related lymphedema by progressive resistance training – a long-term follow-up of the LYCA Randomized Controlled Trial.**

**Journal of Cancer Survivorship**

Gunn Ammitzbøll, Marie Lillegaard, Christoffer Johansen, Charlotte Lanng, Kathrine Grell, Niels Kroman, Bo Zerahn, Ole Hyldegaard, Merete Celano Wittenkamp, Susanne Oksbjerg Dalton.

**Corresponding author**:

Gunn Ammitzbøll

Danish Cancer Institute, Strandboulevarden 49, 2100 Copenhagen, Denmark. Email: gunnam@cancer.dk.

**Supplementary Table 3.** Effect of progressive resistance training on function scales in the EORTC QLQ C30 stratified by the presence of symptom cluster at baseline in women with primary breast cancer treated with axillary lymph node dissection and radiotherapy in the LYCA study, East Denmark.

|  |  | Symptom cluster at baseline NO | | | | Symptom cluster at baseline YES | | | |  |
| --- | --- | --- | --- | --- | --- | --- | --- | --- | --- | --- |
|  | Assessment time | α | 95% CI | p-value | Overall p-value | α | 95% CI | p-value | Overall p-value | Interaction  p-value |
| QL2 | 20 w | -2.8 | -11.5; 5.7 | 0.52 | 0.32 | 8.8 | -0.8; 18.2 | 0.07 | **0.01** | **0.03** |
|  | 12 m | 2.0 | -6.3; 10.1 | 0.64 |  | 12.7 | 3.4; 21.8 | **0.01** |  |  |
|  | 3.5 y | -7.7 | -17.7; 2.0 | 0.12 |  | -0.3 | -10.9; 10.2 | 0.96 |  |  |
| PF | 20 w | 0.1 | -7.7; 7.8 | 0.97 | 0.69 | 7.6 | -1.0; 16.1 | 0.08 | 0.14 | 0.17 |
|  | 12 m | 0.3 | -7.1; 7.6 | 0.93 |  | 6.8 | -1.4; 14.9 | 0.10 |  |  |
|  | 3.5 y | -3.7 | -12.4; 4.9 | 0.40 |  | 3.2 | -6.1; 12.4 | 0.49 |  |  |
| RF | 20 w | -3.9 | -21.3; 13.2 | 0.66 | 0.98 | 8.7 | -10.1; 27.1 | 0.36 | 0.18 | 0.24 |
|  | 12 m | 6.4 | -10.4; 22.9 | 0.44 |  | 17.2 | -1.3; 35.2 | 0.06 |  |  |
|  | 3.5 y | 5.5 | -14.8; 25.4 | 0.59 |  | 4.7 | -16.4; 25.4 | 0.66 |  |  |
| EF | 20 w | 6.7 | -2.8; 16.1 | 0.16 | **0.01** | 13.7 | 3.2; 23.9 | **0.01** | **0.00** | 0.14 |
|  | 12 m | 5.3 | -3.8; 14.2 | 0.24 |  | 15.3 | 5.2; 25.2 | **0** |  |  |
|  | 3.5 y | -11 | -22.2; 0.0 | 0.05 |  | -8.4 | -20.1; 3.1 | 0.15 |  |  |
| CF | 20 w | -0.1 | -13.6; 13.1 | 0.98 | 0.89 | 13.5 | -1.2; 27.9 | 0.07 | 0.01 | 0.22 |
|  | 12 m | 6.9 | -6.2; 19.8 | 0.29 |  | 19.9 | 5.6; 33.8 | **0.01** |  |  |
|  | 3.5 y | 4.4 | -11.5; 20.0 | 0.58 |  | 5.9 | -10.3; 21.7 | 0.47 |  |  |
| SF | 20 w | 9.1 | -8.1; 26.1 | 0.29 | 0.68 | 27.6 | 8.5; 46.3 | **0** | **0.00** | **0.02** |
|  | 12 m | 0.7 | -15.9; 17.0 | 0.93 |  | 26.3 | 7.6; 44.7 | **0.01** |  |  |
|  | 3.5 y | -3.4 | -24.1; 17.0 | 0.75 |  | -1.2 | -22.6; 19.8 | 0.91 |  |  |

α: estimated between group difference for the intervention group compared to the control group. 95% CI: 95% confidence interval. p-value: statistical significance level for between group differences at the specified assessment time. Overall p-value: statistical significance level for between group differences across all assessment times. Interaction p-value: statistical significance level for the interaction between effect and symptom cluster yes/no at baseline. EORTC: European Organisation for Research and Treatment of Cancer. C30: core 30-item scale. QL2: global quality of life subscale. PF: physical functioning subscale. RF: role functioning subscale. EF: emotional functioning subscale. CF: cognitive functioning subscale. SF: social functioning subscale. Symptom cluster score: combined score for the symptom scales of pain, sleep, and fatigue on the EORTC C30. W: weeks. M: months. Y: years. Bold numbers indicate statistical significance level of less than 5% uncertainty.
